# Breed differences in the expression levels of gga-miR-222a in laying hens influenced H_2_S production by regulating methionine synthase genes in gut bacteria

**DOI:** 10.1186/s40168-021-01098-7

**Published:** 2021-08-25

**Authors:** Si-Cheng Xing, Chun-Bo Huang, Rui-Ting Wu, Yi-Wen Yang, Jing-Yuan Chen, Jian-Dui Mi, Yin-Bao Wu, Yan Wang, Xin-Di Liao

**Affiliations:** 1 College of Animal Science, South China Agricultural University, Guangzhou, 510642 China; 2 Guangdong Provincial Key Lab of Agro-Animal Genomics and Molecular Breeding and Key Laboratory of Chicken Genetics, Breeding and Reproduction, Ministry Agriculture, Guangzhou, 510642 Guangdong China; 3 National-Local Joint Engineering Research Center for Livestock Breeding, Guangzhou, 510642 Guangdong China

**Keywords:** miRNA, Metatranscriptome, gga-miR-222a, Hydrogen sulfide, Laying hens

## Abstract

**Background:**

The microbiota in the cecum of laying hens is crucial for host digestion, metabolism, and odor gas production. The results of recent studies have suggested that host microRNAs (miRNAs) can regulate gene expression of the gut microbiota. In the present study, the expression profiles of host-derived miRNAs in the cecal content of two laying hen breeds; Hy-line Gray and Lohmann Pink, which have dissimilar H_2_S production, were characterized; and their effects on H_2_S production by regulating the expression of gut microbiota-associated genes were demonstrated.

**Results:**

The differential expression of microbial serine O-acetyltransferase, methionine synthase, aspartate aminotransferase, methionine-gamma-lyase, and adenylylsulfate kinase between the two hen breeds resulted in lower H_2_S production in the Hy-line hens. The results also revealed the presence of miRNA exosomes in the cecal content of laying hens, and an analysis of potential miRNA-target relationships between 9 differentially expressed miRNAs and 9 differentially expressed microbial genes related to H_2_S production identified two methionine synthase genes, *Odosp_3416* and *BF9343_2953*, that are targeted by gga-miR-222a. Interestingly, in vitro fermentation results showed that gga-miR-222a upregulates the expression of these genes, which increased methionine concentrations but decreased H_2_S production and soluble sulfide concentrations, indicating the potential of host-derived gga-miR-222a to reduce H_2_S emission in laying hens.

**Conclusion:**

The findings of the present study reveal both a physiological role by which miRNAs shape the cecal microbiota of laying hens and a strategy to use host miRNAs to manipulate the microbiome and actively express key microbial genes to reduce H_2_S emissions and breed environmentally friendly laying hens.

**Video Abstract**

**Supplementary Information:**

The online version contains supplementary material available at 10.1186/s40168-021-01098-7.

## Background

The laying hen industry is an important livestock sector that produces eggs, a common source of human nutrition [1]. Nutrient digestion in laying hens is characterized by inadequate enzymatic hydrolysis in the foregut followed by further microbial fermentation in the cecum. An increasing number of studies have demonstrated that this ‘bacterial organ’ plays a crucial role in host metabolism, immunity, and disease [2–4]. Bacteria ferment undigested feed components to generate volatile fatty acids (VFAs), amino acids, ammonia (NH_3_), hydrogen sulfide (H_2_S), and other metabolites [5, 6]. Previous studies elucidated that NH_3_ and H_2_S are the two primary odorous gasses in poultry houses and represent a great loss in nutrients and cause environmental pollution that is a public concern [7, 8]. Although NH_3_, followed by H_2_S, accounts for the largest proportion of odor gas in livestock and poultry facilities, the odor threshold of H_2_S is significantly lower than that of NH_3_ and also contributes to the poor smell in the farm environment [9, 10]. In addition to its adverse effects on air quality, an early study reported that H_2_S is released in the intestines of humans, where it causes a number of intestinal diseases [11]. In a recent study, H_2_S inhalation was shown to induce pneumonia in chickens [12], revealing the potential for H_2_S to damage the health of both workers and animals. Therefore, in addition to mitigating environmental problems, reducing H_2_S levels will have a positive effect on animal health. Several recent studies have explored the effects of nutritional manipulation on H_2_S emission in animals. For example, probiotic inclusion or protein reduction in the diet has been used to regulate the gut microbiota in animals [13, 14]. However, there are a few disadvantages to nutritional manipulations, including the need for the continuous supplementation of probiotics to guarantee the sustained reduction in H_2_S emissions. Thus, the development of breeding laying hens with a low-H_2_S emission “cecal microbiota structure” may represent a better and more permanent solution to promote H_2_S reduction and an environmentally friendly culture in the poultry industry. However, to breed low-H_2_S emission laying hens, it is first important to understand the regulatory relationship between the host and its cecal microbiota.

Numerous factors influence the composition and function of the gut microbiota, such as the genetic background of the host, and to some extent, these factors can be transiently altered by diet, the environment, and disease states [15, 16]. MicroRNAs (miRNAs) are a group of noncoding RNAs ~ 22 nucleotides (nt) in length that are known for their sequence-specific regulatory function that involves targeting the 3′ untranslated region of mRNAs in the cytoplasm [17]. Increasing evidence has demonstrated that miRNAs also exist extracellularly and circulate in body fluids within exosomes and microvesicles. Secreted miRNAs have been isolated from blood, milk, and even stool and urine [18–22], with some studies having characterized miRNAs as potential markers of tumorigenesis in human stool [23–25]. Interestingly, recent studies have also revealed that host-derived miRNAs can serve as an important crosstalk channel between the host and the intestinal bacterial population. Liu et al. demonstrated that the host can modulate the gut microbiota through intestinal epithelial cell-secreted miRNAs, which enter gut bacteria and directly regulate bacterial gene expression [26]. Other studies have reported that plant-derived miRNAs can be taken up by gut bacteria and shape the gut microbiota [27, 28]. Therefore, we hypothesized that cross-regulation may occur between host-derived miRNAs and the cecal microbiota in laying hens. In addition, it remains unknown whether host-derived miRNAs regulate microbial abundance or the expression of bacterial functional genes, changes in which influence the structure and metabolic functions of the microbiota, and potentially lead to H_2_S production differences in different breeds of laying hens.

In our previous study, we observed a significantly higher daily H_2_S production in Lohmann laying hens than in Hy-line Gray laying hens (daily H_2_S production per kg average daily feed intake was 7.75 and 4.17 mg, respectively) [29]. However, whether this difference in H_2_S emissions was due to host miRNA regulation of the gut microbiota remained unelucidated. Therefore, in the present study, we determined the expression profiles of host-derived miRNAs in the cecal contents of these two breeds of laying hens to identify miRNAs showing significantly different expression and then predicted the target relationships between differentially expressed miRNAs and microbial genes related to H_2_S production. Finally, the effects of selected targeted miRNAs on H_2_S production in laying hens were verified through in vitro experiments. The results of the present study should provide a better understanding of the interkingdom regulatory relationships among miRNAs, cecal microbiota and H_2_S production in laying hens, providing a reference for the breeding of environmentally friendly laying hens. At the same time, miRNAs, which have been proven to regulate the production of H_2_S in the cecum of laying hens, could be used as a safe and clean additive to promote reductions in H_2_S emissions in the future.

## Results

### Determination of miRNA profiles in the cecum of laying hens

**Fig. 1:**
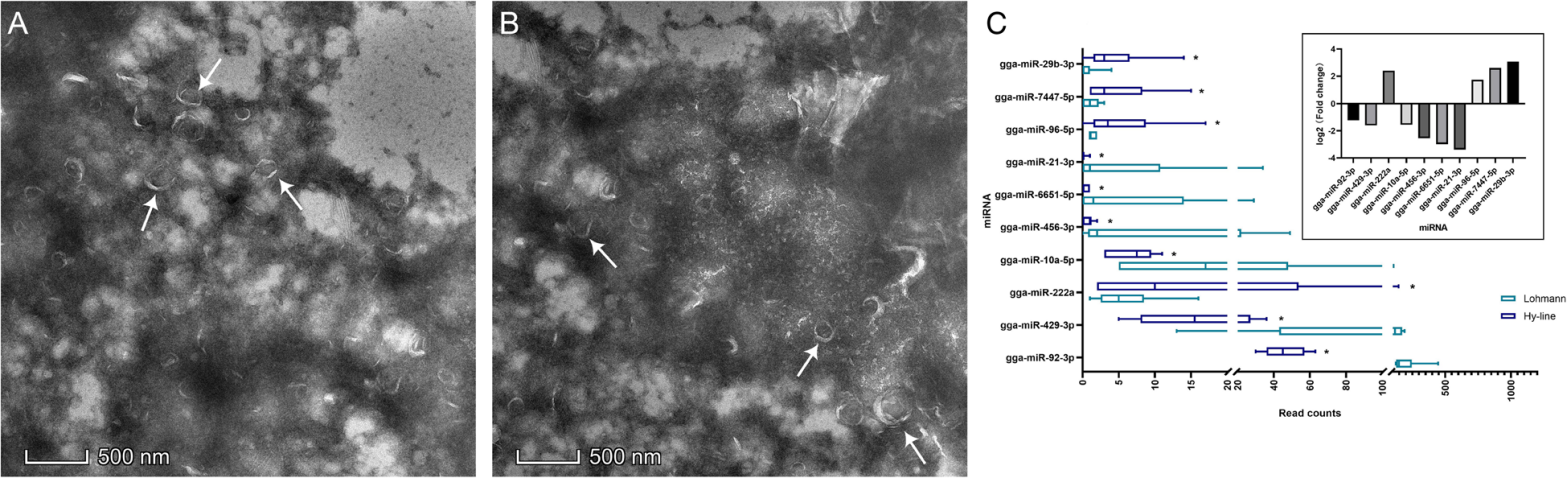
Morphological characteristics of exosomes and significantly expressed miRNAs. Morphological characteristics of exosomes in Lohmann cecal contents (**A**) and in Hy-line cecal contents (**B**). White arrows indicate exosomes. **C** Ten differentially expressed miRNAs between the two breeds. Asterisk indicates a significant difference in an miRNA between the two breeds, and significant differences between the means were determined by Tukey’s test. Differences were considered significant at *P* < 0.05, and the bar graph in **C** shows the expression level of these 10 miRNAs. The bars on the “0” X axis indicate upregulation in the Hy-line hens compared to the Lohmann hens, and the bars under the “0” X axis indicate downregulation in the Hy-line hens compared to the Lohmann hens

The morphology of exosomes derived from laying hen cecal contents was observed by transmission electron microscopy (TEM). Exosome-sized (approximately 50–200 nm in diameter) extracellular vesicles were present in the cecal contents of Lohmann and Hy-line hens, but their morphological characteristics were largely the same in the two breeds (Fig. 1A, B). Based on the observed presence of exosomes, miRNA sequencing was performed to investigate the miRNA differences between the two breeds.

From the high-throughput sequencing results, we identified 288 annotated miRNAs using miRBase, the associated information for which is presented in Table S1. Subsequently, a DEGSeq analysis using the R software environment revealed 10 miRNAs as being significantly differentially expressed between the two breeds, where the log2 (fold change) > 1 results identified four miRNAs (gga-miR-222a, gga-miR-96-5p, gga-miR-7447-5p, and gga-miR-29b-3p) with significantly higher expression levels and six miRNAs (gga-miR-92-3p, gga-miR-429-3p, gga-miR-10a-5p, gga-miR-456-3p, gga-miR-6651-5p, and gga-miR-21-3p) showing significantly lower expression levels in the Hy-line hens than in the Lohmann hens (*P* < 0.05). In addition, the top four highly expressed miRNAs were gga-miR-92-3p (Hy-line: 46 ± 11.47; Lohmann, 196 ± 113.67), gga-miR-429-3p (Hy-line, 17.5±10.5; Lohmann, 110.167 ± 61.50), gga-miR-222a (Hy-line, 32.167 ± 51.547; Lohmann, 6 ± 4.80), and gga-miR-10a-5p (Hy-line, 6.833 ± 3.08; Lohmann, 30 ± 35.81) in Hy-line and Lohmann (Fig. 1C). Thus, the results indicated that these four miRNAs should be considered for further analysis.

The KEGG pathway annotations of target genes in the chicken genome for these ten significantly expressed miRNAs are shown in Fig. S1 and were mostly enriched in metabolic pathways, neuroactive ligand-receptor interaction, focal adhesion, endocytosis and purine metabolism.

### Expression of microbial genes related to H_2_S production

**Fig. 2:**
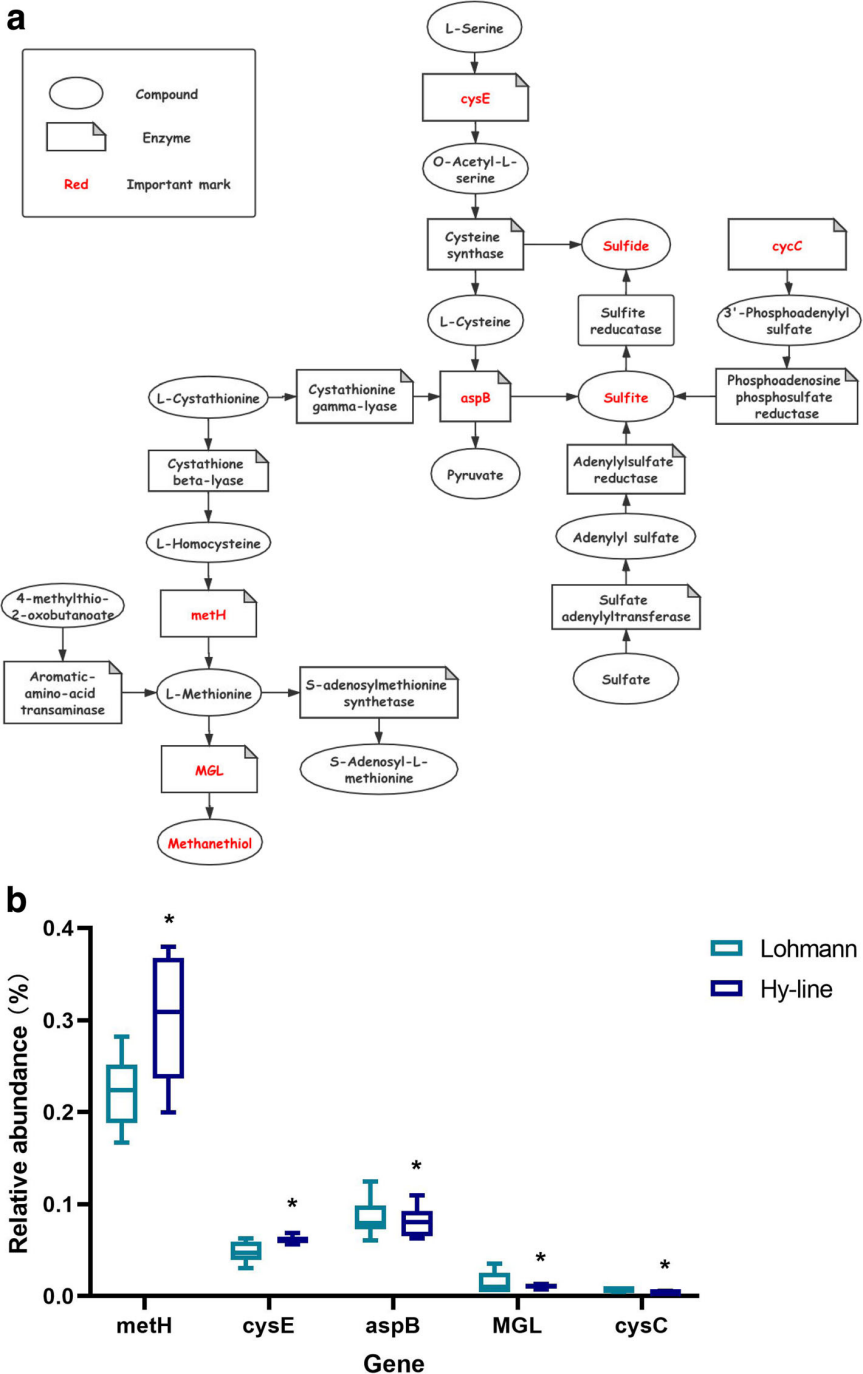
Expression of genes encoding enzymes in the cysteine and methionine metabolism and sulfate metabolism pathways. **A** Enzymes and intermediates involved in the cysteine and methionine metabolic pathway and that of sulfate. **B** Differentially expressed genes that are marked in red in metabolic pathways. Asterisk indicates a significant difference in relative gene abundance between the two breeds, where significant differences between the means were determined with Tukey’s test. Differences were considered significant at *P* < 0.05

The sequencing information for the metatranscriptome is listed in Table S2, and the top 30 KEGG microbial function enrichment pathways are shown in Fig. S2. The 5 pathways showed significant differences in gene enrichment between the Lohmann and Hy-line hens, where the abundances of microbial genes associated with the two-component system were significantly higher in the Hy-line hens than in the Lohmann hens (*P* < 0.05), and the abundances of microbial genes enriched in the amino sugar and nucleotide sugar metabolism, cysteine and methionine metabolism, alanine aspartate and glutamate metabolism, and RNA degradation pathways were significantly higher in Lohmann hens than in the Hy-line hens (*P* < 0.05). The transcriptomics results also showed that the abundances of 22,237 genes were significantly different between the Lohmann and Hy-line hens (*P* < 0.05). In addition, based on the combined analysis of the microbial gene enrichment pathways and significantly differentially expressed genes, we focused on two metabolic pathways involved in H_2_S production, the cysteine and methionine metabolism pathway (map 00270) and the sulfur metabolism pathway (map 00920). Based on the KEGG database, the degradation of sulfur-containing amino acids, cysteine, and methionine, can result in the production of H_2_S. As shown in Fig. 2A, the synthesis of L-cysteine from L-serine and sulfide is catalyzed by serine O-acetyltransferase and cysteine synthase, and L-cysteine can also be synthesized from L-cystathionine by cystathionine gamma-lyase. L-cysteine is degraded into pyruvate and sulfite by aspartate aminotransferase, and sulfite is used as a raw material for H_2_S production in the sulfur metabolism pathway. In addition, methionine can be degraded into methanethiol by methionine-gamma-lyase, which can then be converted into H_2_S in subsequent processes. Another pathway for methionine degradation involves the formation of S-adenosyl-L-methionine by S-adenosylmethionine synthetase without H_2_S production. Sulfur metabolism is an essential pathway in the cecum of hens and is involved in the production of H_2_S. As the assimilatory reduction and dissimilatory reduction of sulfate promotes the production of sulfite and its eventual conversion into sulfide (Fig. 2A), adenylylsulfate kinase is a key enzyme for the production of sulfide from sulfate and was further evaluated to elucidate H_2_S production in hens.

By analyzing the annotation information in the KEGG database, the results indicated that O-acetyltransferase (*cysE*, EC:2.3.1.30), methionine synthase (*metH*, EC:2.1.1.13), aspartate aminotransferase (*aspB*, EC:2.6.1.1), methionine-gamma-lyase (*MGL*, EC:4.4.1.11) and adenylylsulfate kinase (*cysC*, EC: 2.7.1.25) are the key enzymes for the production of H_2_S. Thus, the gene expression levels of these key enzymes were next tested. The expression levels of both *cysE* and *metH* were significantly higher in the Hy-line hens than in the Lohmann hens (*P* < 0.05); the expression level of *cysE* was 0.061 ± 0.004 and 0.048 ± 0.010 in the Hy-line and Lohmann hens, respectively; and the expression level of *metH* was 0.302 ± 0.062 and 0.222 ± 0.036 in the Hy-line and Lohmann hens, respectively. In contrast, the expression levels of both *aspB* and *MGL* were lower in the Hy-line hens than in the Lohmann hens (*P* < 0.05); the expression level of *aspB* was 0.081 ± 0.015 and 0.085 ± 0.020 in the Hy-line and Lohmann hens, respectively; and the expression level of *MGL* was 0.011 ± 0.002 and 0.014 ± 0.011 in the Hy-line and Lohmann hens, respectively. These results indicated that there was a higher tendency for the synthesis of cysteine and methionine but a lower tendency for their degradation and H_2_S production in the cecum of Hy-line hens (Fig. 2B). In addition, we also observed significantly lower *cysC* expression levels in the cecum of Hy-line hens than in Lohmann hens (*P* < 0.05; 0.004 ± 0.001 and 0.007 ± 0.001, respectively). This result also indicated that less sulfide is produced in Hy-line hens than in Lohmann hens (Fig. 2B).

Based on an analysis of the regulatory enzymes involved in H_2_S production, thirteen differentially expressed microbial genes involved in encoding these five enzymes were identified between the two breeds. *MGL* and *cysC* were assigned to only one gene and one genus, and the other three enzymes were assigned to several genes and genera. In addition, most of the genes were assigned to the genus *Bacteroides* (Table 1).

**Table 1:** Differentially expressed genes (DEGs) and source bacteria of differentially expressed enzymes

Enzymes	DEGs (log2 Fold change)	Source bacteria
*CysE*	Ddes_0279 (6.35)	*Desulfovibrio desulfuricans*
	CK3_01000 (5.31)	*Unclassified Clostridiales*
	SELR_06150 (4.94)	*Selenomonas ruminantium*
	Bache_0784 (2.36)	*Bacteroides helcogenes*
*MetH*	Odosp_3416 (8.48)	*Odoribacter splanchnicus*
	Bacsa_0021 (5.67)	*Bacteroides salanitronis*
	BF9343_2953 (5.31)	*Bacteroides fragilis* NCTC9343
*AspB*	BVU_0144 (− 5.37)	*Bacteroides vulgatus*
	Bache_2087 (− 4.20)	*Bacteroides helcogenes*
	PRU_1300 (− 3.66)	*Prevotella ruminicola*
	OBV_25710 (− 3.27)	*Oscillibacter valericigenes*
*MGL*	GFO_2175 (− 5.68)	*Gramella forsetii*
*CysC*	Mmc1_2549 (− 4.76)	*Magnetococcus marinus*

### Target prediction of miRNAs

**Fig. 3:**
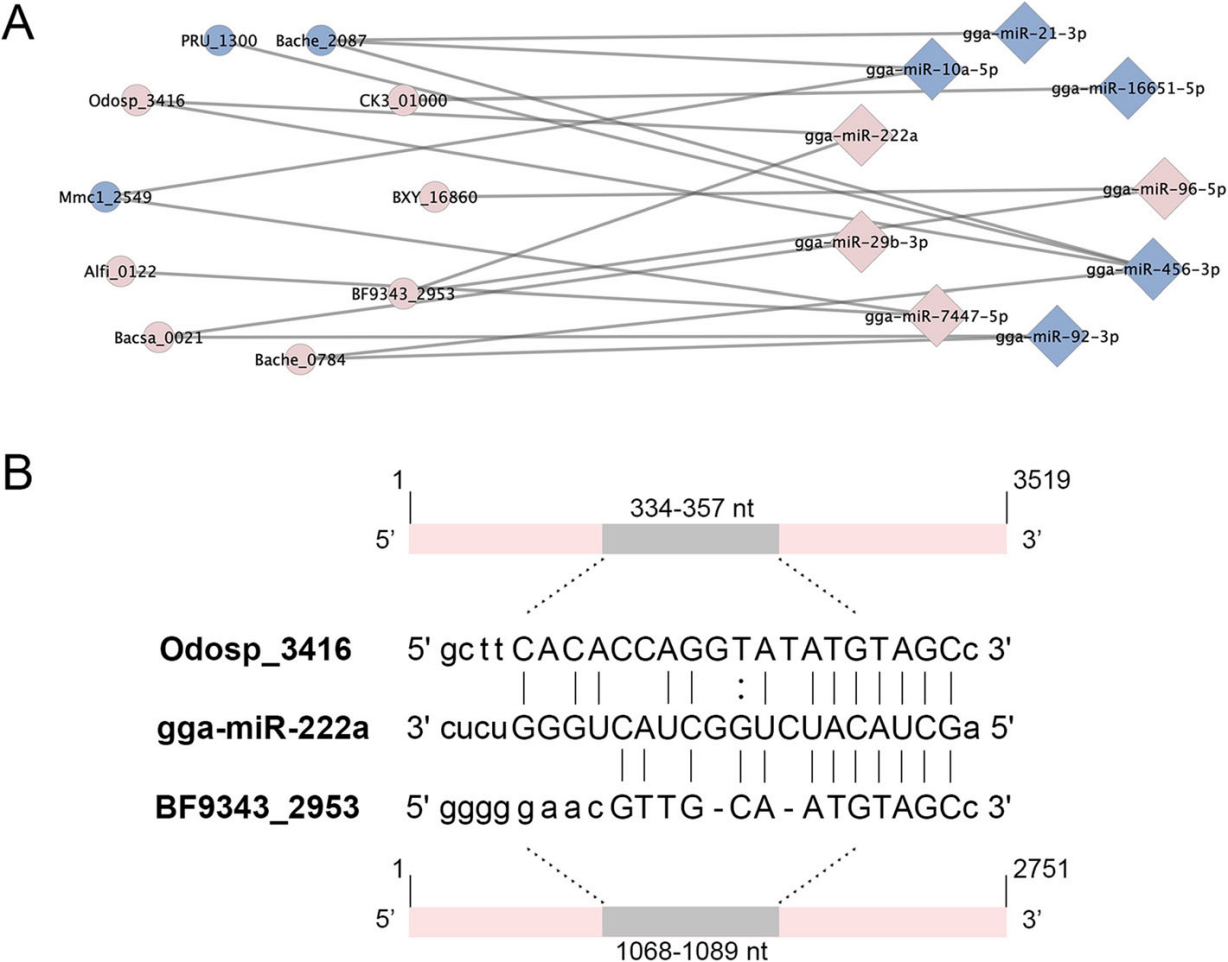
Correlation and target site analysis between miRNAs and bacterial genes. **A** Significantly upregulated genes and miRNAs in the Hy-line hens compared to the Lohmann hens (pink); significantly downregulated genes and miRNAs in the Hy-line compared to the Lohmann hens (blue). Squares indicate bacterial genes, and circles indicate host miRNAs. **B** Target sites between gga-miR-222a and the genes *Odosp_3416* and *BF9343_2953*

Through a combination analysis of differentially expressed miRNAs (Fig. 1C) and differentially expressed genes associated with H_2_S production (Table 1), nine miRNAs were identified as being able to target 9 genes through miRanda analysis (Fig. 3A). As mentioned above, gga-miR-222a and gga-miR-10a-5p were both highly expressed miRNAs in Hy-line and Lohmann hens, although the abundance of gga-miR-222a was significantly higher in Hy-line hens than in Lohmann hens, while the abundance of gga-miR-10a-5p was significantly lower in Hy-line hens than in Lohmann hens. In addition, the results of our previous study showed that the amount of H_2_S production was obviously higher in Lohmann hens than in Hy-line hens (daily H_2_S production per kg average daily feed intake of 7.75 and 4.17 mg, respectively) [29]. Therefore, we concluded that gga-miR-222a is a potential candidate additive for use in the reduction of H_2_S production in the cecum of laying hens and should be further investigated. We observed that gga-miR-222a could target two genes associated with methionine synthase, *Odosp_3416* and *BF9343_2953*, expressed by the bacteria *Odoribacter splanchnicus* and *Bacteroides fragilis* NCTC 9343, respectively (Fig. 3B). The read counts for *Odosp_3416* were 138 and 225 in the Hy-line and Lohmann hens, respectively, while those observed for *BF9343_2953* were 75 and 137. Based on these results, we suspected that the regulatory effect of gga-miR-222a on the two genes may be notable. Therefore, functional verification of gga-miR-222a targeting *Odosp_3416* and *BF9343_2953* was performed.

### H_2_S production during fermentation

After 24 h of fermentation, the amount of total gas and H_2_S production in the different groups was tested, the results of which showed that the addition of gga-miR-222a could influence total gas and H_2_S production.

**Fig. 4:**
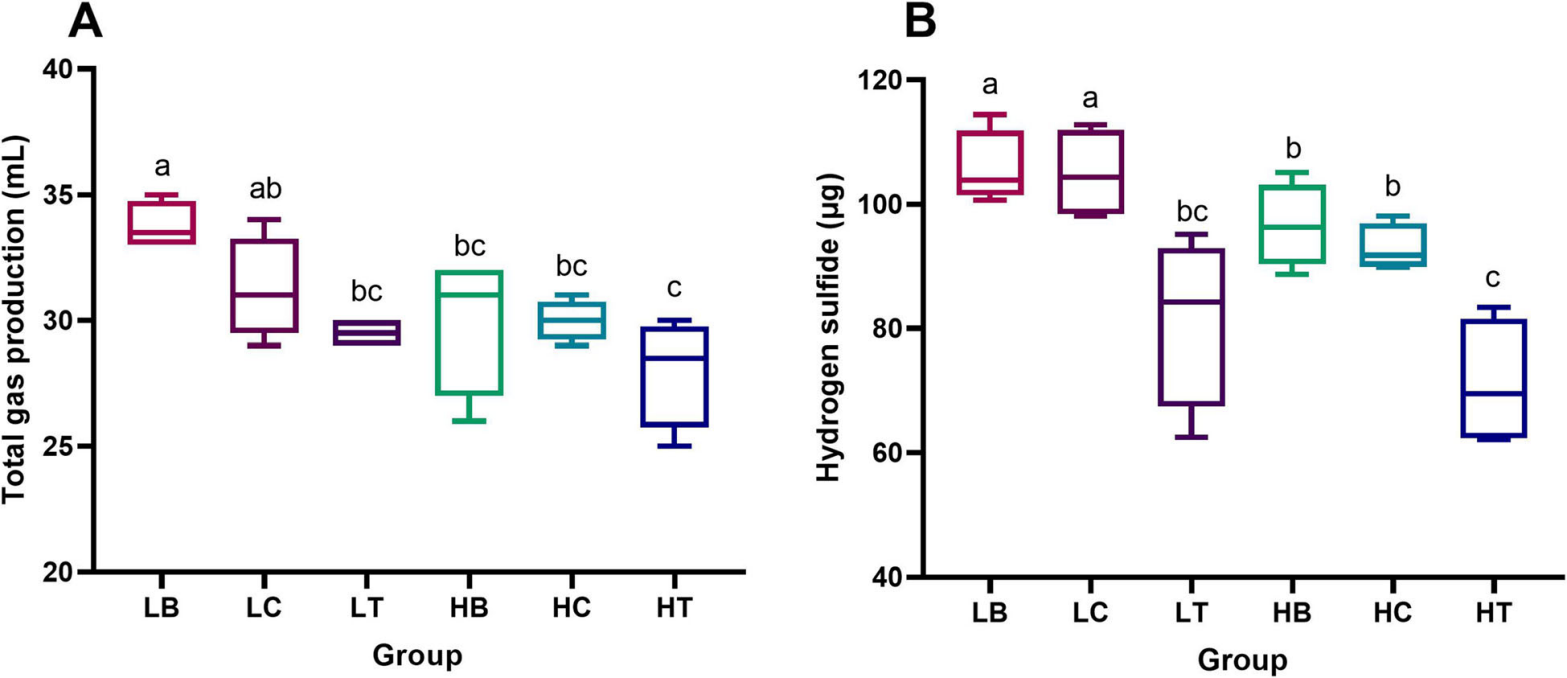
Amount of total gas and H_2_S production in the fermentation experiment for each group. **A** Amount of total gas production and **B** H_2_S production. Different letters indicate significant differences in the amount of total gas and H_2_S production among the groups, and significant differences among the means were determined by Tukey’s test. Differences were considered significant at *P* < 0.05

The total gas production was 33.75 ± 0.83 and 30 ± 0.71 mL in the Lohmann intestinal content broth with nothing added (LB) and Hy-line intestinal content broth with nothing added (HB), respectively. The statistical analysis showed that the total gas production in the LB group was significantly higher than that in the HB group (*P* < 0.05), potentially indicating that the total gas production ability of Lohmann hens was higher than that of Hy-line hens. In the present study, we observed that the amount of total gas production was significantly decreased in the Lohmann intestinal content broth supplemented with gga-miR-222a (LT; 29.5 ± 0.5 mL) compared to the LB group (*P* < 0.05); However, no significant difference was observed between the Hy-line intestinal content broth supplemented with gga-miR-222a (HT; 28 ± 1.87 mL) and HB groups. These results demonstrated that gga-miR-222a can effectively decrease the total gas production in Lohmann hens compared to Hy-line hens (Fig. 4A). In addition, no obvious difference was observed between the blank groups (intestinal content broth without the addition of miRNA, groups LB and HB) and the control groups (intestinal content broth with added miRNA control, groups LC and HC), suggesting that the result obtained with the commercially synthesized gga-miR-222a were credible.

The effect of gga-miR-222a on H_2_S production was investigated, the results of which are shown in Fig. 4B. The amount of H_2_S was 105.747 ± 5.22 and 96.592 ± 5.84 μg in the LB and HB groups, respectively, and the statistical analysis showed that the amount of H_2_S was significantly higher in the LB group than the HB group (*P* < 0.05), indicating that the ability of Lohmann hens to produce H_2_S gas was higher than that of the Hy-line hens. It is worth noting that gga-miR-222a addition significantly decreased the amount of H_2_S production in the fermentation broths of both breeds (*P* < 0.05), and the amount of H_2_S production was 81.553 ± 11 and 71.152 ± 8.94 μg in the LT and HT groups, respectively. Compared to the blank groups, gga-miR-222a addition decreased H_2_S production by 22.88 and 26.33% in the Lohmann and Hy-line intestinal content broths, respectively. These results suggest that gga-miR-222a has the potential to decrease H_2_S production in the intestine.

The chemical indexes of the fermentation broth of each group are shown in Table 2. The concentration of soluble sulfide (S^2−^) was significantly lower in the HB group (12.32 ± 0.98 μg/g) than in the LB group (15.06 ± 0.94 μg/g) (*P* < 0.05). In addition, the S^2−^ concentration was also significantly lower in the gga-miR-222a addition groups (LT, 12.50 ± 0.50 μg/g; HT, 9.84 ± 1.44 μg/g) than in the blank groups (LB and HB) (*P* < 0.05). The concentration of methionine in the fermentation broth was also evaluated, as methionine is the crucial amino acid that donates sulfur to form H_2_S through microbial metabolism. In the present study, the addition of gga-miR-222a also significantly increased the concentration of methionine in the LT (280.39 ± 5.78 μg/mL) and HT (299.12 ± 3.68 μg/mL) groups compared to the LB (246.21 ± 18.83 μg/mL) and HB (257.70 ± 19.30 μg/mL) groups, respectively (*P* < 0.05).

**Table 2:** The comparison of fermentation incubation indexes. Significant differences between the means were determined by Tukey’s test. Differences were considered significant at *P* < 0.05

Items	LB	LC	LT	HB	HC	HT
pH	7.57 ± 0.08	7.49 ± 0.03	7.61 ± 0.10	7.59 ± 0.02	7.55 ± 0.05	7.48 ± 0.05
S^2−^, μg/g	15.06 ± 0.94^a^	14.83 ± 1.12^a^	12.50 ± 0.50^b^	12.32 ± 0.98^b^	12.99 ± 1.59^ab^	9.84 ± 1.44^c^
SO_4_^2−^, mg/g	256.94 ± 29.93	268.52 ± 61.26	221.30 ± 79.04	257.87 ± 85.95	287.96 ± 46.39	243.98 ± 70.84
Acetate, mmol/L	31.53 ± 4.01	30.19 ± 0.96	32.81 ± 3.25	32.45 ± 1.85	34.63 ± 2.23	34.15 ± 2.70
Propionate, mmol/L	15.51 ± 1.54	15.34 ± 3.40	15.21 ± 1.35	16.00 ± 1.00	16.58 ± 0.91	15.59 ± 0.74
Butyrate, mmol/L	8.33 ± 0.54^c^	8.16 ± 0.56^c^	9.10 ± 0.73^bc^	10.81 ± 1.44^a^	10.67 ± 1.22^a^	10.17 ± 0.60^ab^
Total VFAs, mmol/L	55.37 ± 6.00	53.69 ± 3.18	57.11 ± 5.29	59.26 ± 2.94	61.88 ± 1.98	59.91 ± 3.70
Methionine, μg/mL	246.21 ± 18.83^c^	245.88 ± 15.01^c^	280.39 ± 5.78^ab^	257.70 ± 19.30	258.92 ± 12.08	299.12 ± 3.68^a^

Data are presented as means with their standard errors

Means within a row with different superscript letters differ (*P* < 0.05)

*LB* Lohmann intestinal content broth added nothing, *LC*s Lohmann intestinal content broth added miRNA control, *LT* Lohmann intestinal content broth added gga-miR-222a, *HB* Hy-line intestinal content broth added nothing, *HC* Hy-line intestinal content broth added miRNA control, *HT* Hy-line intestinal content broth added gga-miR-222a

### Bacterial abundance and gene expression in fermentation broth

**Fig. 5:**
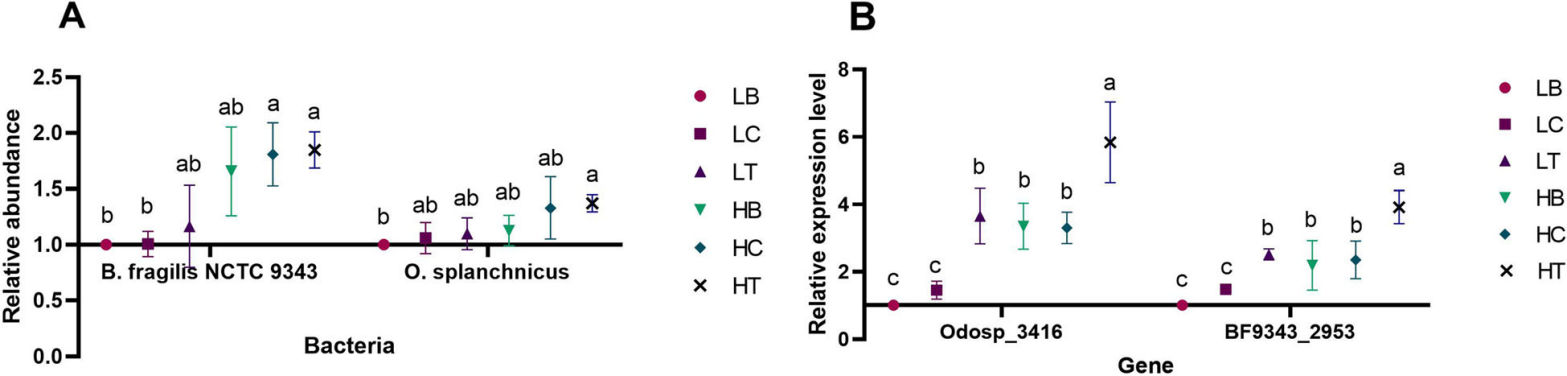
Relative bacterial abundances and gene expression levels in the fermentation experiment for each group. **A** Relative abundances of *Odoribacter splanchnicus* and *Bacteroides fragilis* NCTC9343 in each group. The relative abundances of the LC, LT, HB, HC, and HT groups are shown compared to that of the LB group (set as 1). **B** Relative expression levels of the genes *Odosp_3416* and *BF9343_2953* in each group. The relative abundances of the LC, LT, HB, HC, and HT groups are shown relative to that of LB group (set as 1). Different letters in different groups indicate a significant difference, and significant differences among the means were determined by Tukey’s test (*P* < 0.05).

The methionine synthetase genes (*metH*) *Odosp_3416* (expressed by the bacterium *Odoribacter splanchnicus*) and *BF9343_2953* (expressed by the bacterium *Bacteroides fragilis* NCTC 9343) were predicted to be targeted by gga-miR-222a (Table 1). The abundances of *Odoribacter splanchnicus* and *Bacteroides fragilis* NCTC 9343 and the levels of *Odosp_3416* and *BF9343_2953* expression were quantified to assess the effect of gga-miR-222a on bacterial abundance and gene expression (Fig. 5). The addition of gga-miR-222a had no effect on the abundances of the two bacteria in the intestinal content broth of the two breeds when comparing the blank groups (Fig. 5A). In contrast, the expression of the genes *Odosp_3416* and *BF9343_2953* in the LT (*Odosp_3416*, 3.648 ± 0.72; *BF9343_2953*, 2.513 ± 0.14) and HT (*Odosp_3416*, 5.837 ± 1.04; *BF9343_2953*, 3.918 ± 0.42) groups was significantly higher than that observed in the LB (*Odosp_3416*, 1 ± 0; *BF9343_2953*, 1 ± 0) and HB (*Odosp_3416*, 3.353 ± 0.59; *BF9343_2953*, 2.199 ± 0.64) groups (*P* < 0.05) (Fig. 5B). These results suggest that gga-miR-222a increases *metH* (*Odosp_3416* and *BF9343_2953*) expression in the intestinal content broths of the two breeds. However, the results also showed that gga-miR-222a did not significantly affect the abundances of target gene-related bacteria.

### Efficacy of gga-miR-222a treatment

**Fig. 6:**
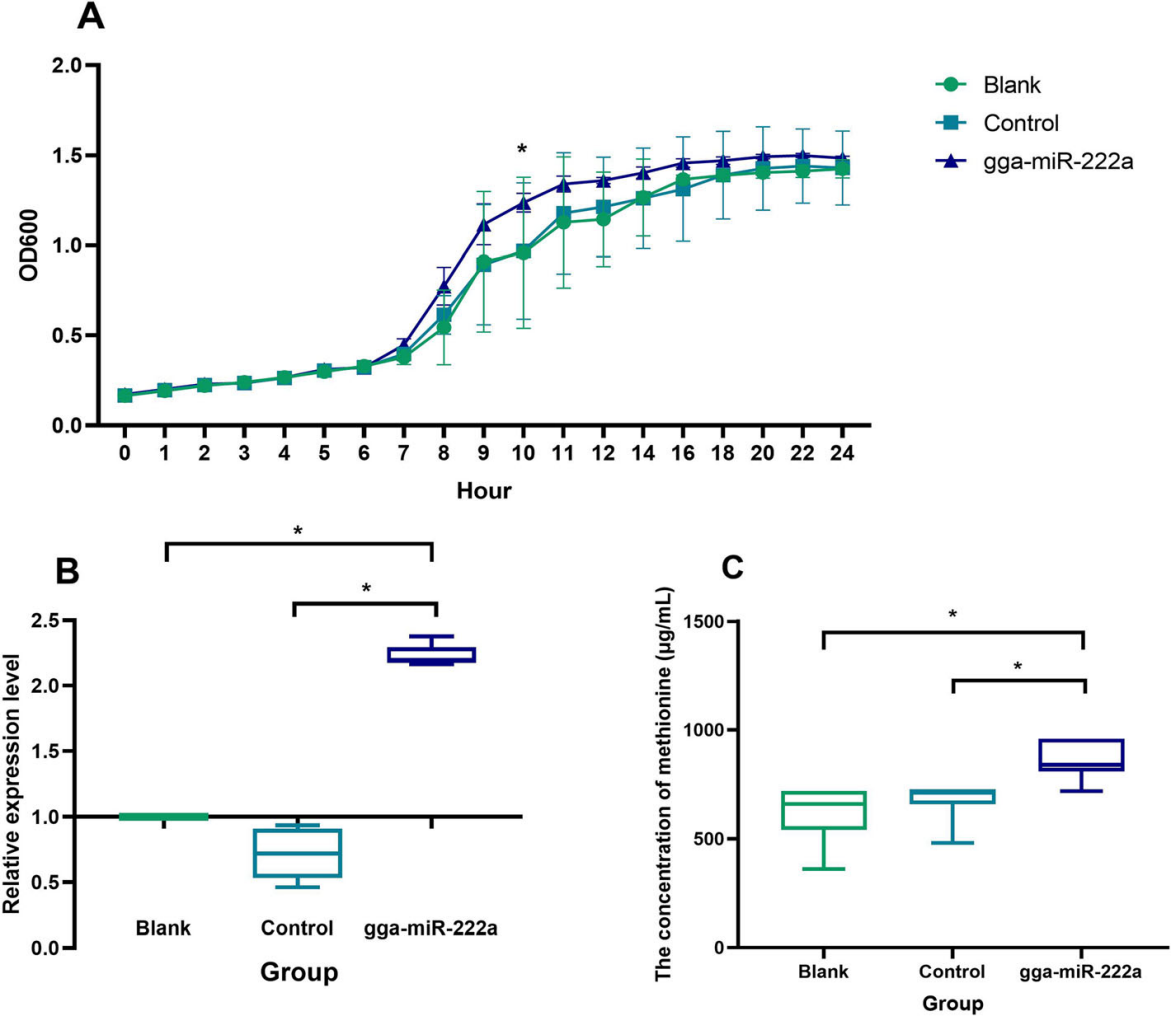
Effects of gga-miR-222a on the growth and metabolism of *Bacteroides fragilis* NCTC9343. **A** Growth curve of *Bacteroides fragilis* NCTC9343. **B** Expression of the *BF9343_2953* gene in bacterial culture medium. The relative abundances of the control and gga-miR-222a groups are shown compared to that of the blank group (set as 1). **C** Methionine concentration in the bacterial culture medium. In the figures, asterisk indicates a significant difference among groups, and significant differences among the means were determined by Tukey’s test (*P* < 0.05)

To further understand whether the effects of gga-miR-222a truly influence the methionine synthetase genes-harboring bacteria, a culture medium experiment was performed. Because the relative abundance of *Odoribacter splanchnicus* was significantly lower than that of *Bacteroides fragilis* NCTC9343 in both hen breeds (Fig. S3), *Bacteroides fragilis* NCTC9343 was selected for the subsequent experiment. At 8 and 10 h, the bacterium *Bacteroides fragilis* NCTC9343 reached the logarithmic and plateau phases, respectively. In the gga-miR-222a addition group, this miRNA significantly improved (29.04%) the abundance of *Bacteroides fragilis* NCTC9343 at 10 h compared to the blank (*P* < 0.05) (Fig. 6A). In addition, gga-miR-222a addition also significantly enhanced the expression of the *Bacteroides fragilis* NCTC9343 gene *BF9343_2953* by 2.23-fold compared to the control (*P* < 0.05) (Fig. 6B), and the concentration of methionine in the medium was also significantly increased (38.71%) with the addition of gga-miR-222a compared to the blank (*P* < 0.05) (Fig. 6C).

**Fig. 7:**
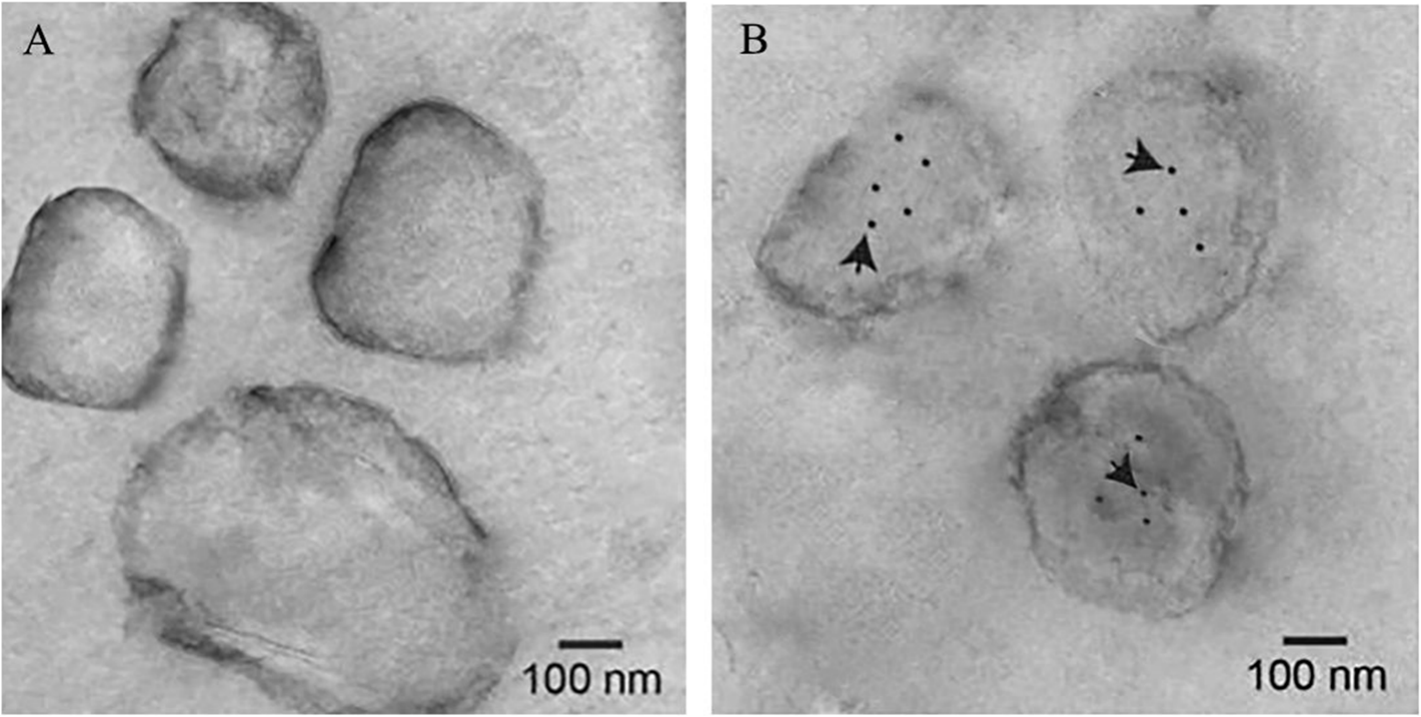
In situ hybridization map of gga-miR-222a internalized in *Bacteroides fragilis* NCTC9343. In situ hybridization map, where **A** is the miRNA control group and **B** is the gga-miR-222a group. Black dots and arrows indicate the site of hybridization

To determine whether gga-miR-222a could be taken up by and exhibit regulatory functions in *Bacteroides fragilis* NCTC9343, we measured the bacterial internalization of gga-miR-222a by in situ hybridization and TEM. Compared to the control (Fig. 7A), exogenous gga-miR-222a was selectively absorbed by *Bacteroides fragilis* NCTC9343 in the gga-miR-222a addition group (Fig. 7B).

## Discussion

**Fig. 8:**
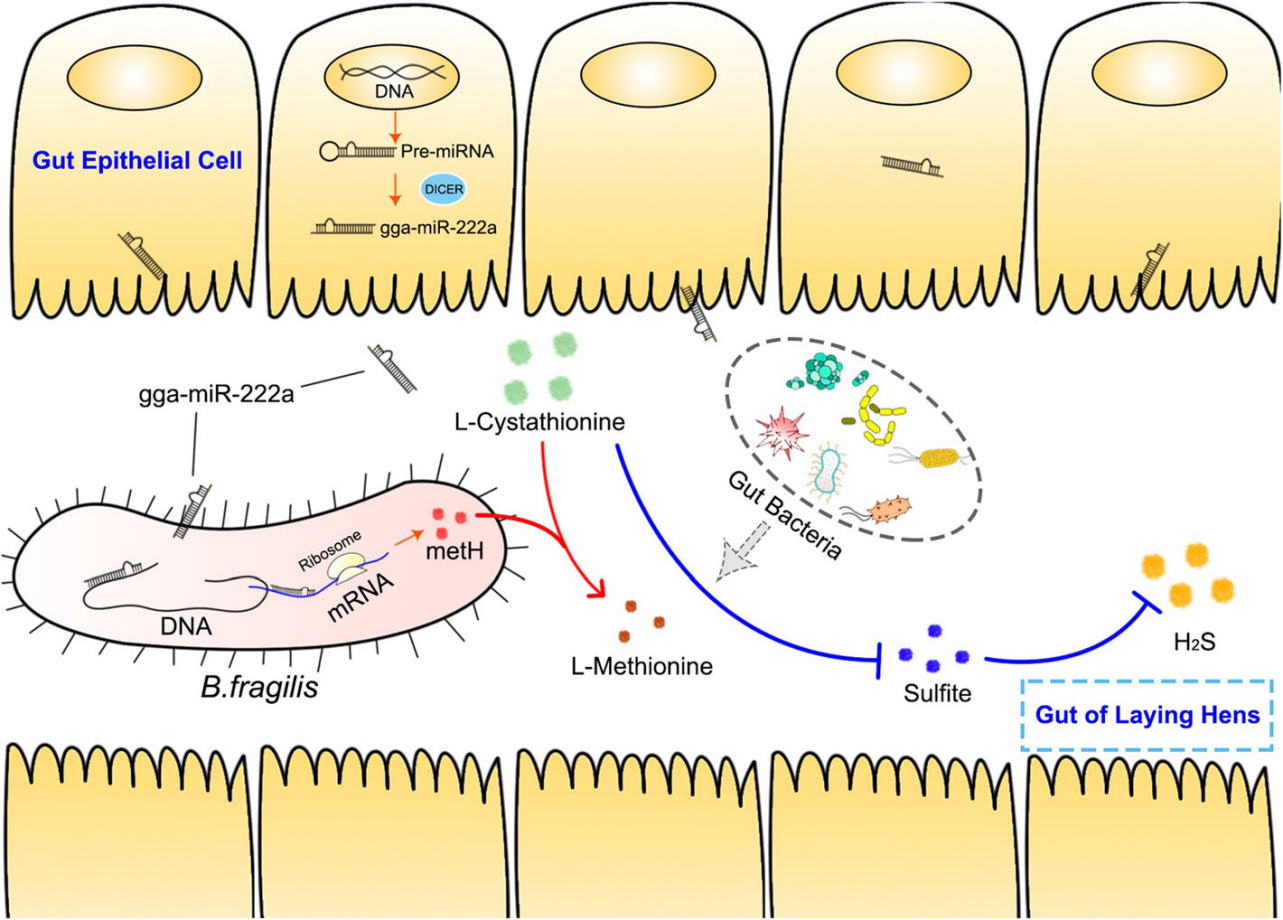
The involving pathway of gga-miR-222a reduce the production of H_2_S in the intestine of laying hens

Although there is a known association among host genetic background, cecal microbiota structure, and odor production by laying hens [30, 31], the potential mediators of this relationship remain unclear. Recently, a number of findings have demonstrated that mammals such as mice secrete miRNAs that could regulate gene transcripts in bacteria (such as *F. nucleatum* and *E. coli*) and affect bacterial growth [26, 32]. In the present study, we presented the first characterization of miRNAs derived from the cecal contents of laying hens and observed that gga-miR-222a can reduce the production of H_2_S by regulating the expression of cecal microbial methionine synthetase genes in the cecum of laying hens (Fig. 8).

Differential expression of cecal microbial genes led to dissimilar H_2_S production between the two hen breeds

In a previous study, we demonstrated that Hy-line hens exhibit lower H_2_S production than Lohmann hens due to different microbiota structures related to H_2_S production in the cecum [29]. However, due to the limitation of 16S rRNA sequencing, we did not annotate and identify pathways and genes related to bacterial sulfur metabolism. Transcriptomic sequencing can more accurately elucidate the functional makeup of a microbial community than other methods and allow potential miRNA interactions to be characterized across the microbiome and transcriptome. The gene expression of the cecal microbiota was characterized with respect to H_2_S production-related pathways through a metatranscriptome analysis. The synthesis of cysteine and methionine requires the participation of sulfur, and its related decomposition is accompanied by the release of sulfur [33]. Higher serine O-acetyltransferase and methionine synthase expression but lower of aspartate aminotransferase and methionine-gamma-lyase expression in the Hy-line cecal microbiota community indicated that the Hy-line hens had a stronger ability to utilize sulfur for the synthesis of cysteine and methionine than the Lohmann hens.

Dissimilatory sulfate reduction is the exclusive sulfate reduction pathway for most sulfate-reducing bacteria (SRB), but assimilatory sulfate reduction can be carried out by most bacteria in the gut [34, 35]. The metatranscriptome results showed that there were no significant differences in the expression of dissimilatory sulfate reduction pathway-related genes, possibly due to the abundance of SRB being low in the animal gut (approximately 0.028–0.097%) [14]. Low abundances of gut SRB led to low and unobvious differential expression of related genes. However, with respect to the assimilatory sulfate reduction pathway, the gene expression of adenylylsulfate kinase in the Hy-line hens was significantly lower than that observed in the Lohmann hens, indicating a greater transformation of sulfate to sulfide in the latter case.

In the present study, we observed that the differentially expressed microbial genes in sulfur-related metabolic pathways led to dissimilar H_2_S production between the Lohmann and Hy-line hens, but whether the host specifically regulates microbial genes by some factors involved in cross-regulation remains unclear. In the present study, we identified cecal miRNAs and showed that they can directly regulate specific bacterial gene expression and affect gut microbial growth to affect H_2_S production in laying hens.

### Cecal content miRNAs differ between the two laying hen breeds

miRNAs have not been previously characterized in the cecal content of laying hens. First, we demonstrated that microvesicles exist in the cecal contents of Lohmann and Hy-line hens, but only 288 known miRNAs were sequenced. Because the bacterial RNA accounted for the primary proportion of total RNA in the cecal contents of laying hens, and since some miRNAs may be degraded by the high temperature and high uric acid environment of the cecum [36], the number of types and abundances of sequenced miRNAs was relatively low. Only 10 miRNAs were differentially expressed between the Lohmann and Hy-line hens. The highly conserved and homologous characteristics of miRNAs may lead to a high similarity in miRNA types and abundances between two breeds [37]. Most of the chicken genome targets of these miRNAs were enriched in metabolic pathways, neuroactive ligand-receptor interaction, focal adhesion, endocytosis, and purine metabolism but not pathways related to cancer occurrence and disease formation, indicating that these miRNAs did not have a potentially negative effect on the normal life activities of the host. These results indicate that the use of these miRNAs in odor reduction may be harmless to the host itself.

### Host-derived miRNAs target the genes of the cecal microbiota of laying hens

miRNAs bind to mRNAs to perform their regulatory functions. We predicted the possible target relationships between differentially expressed miRNAs and differentially expressed microbial genes related to H_2_S production. The results revealed that gga-miR-222a targets the methionine synthetase genes *Odosp_3416* and *BF9343_2953* (expressed by *Odoribacter splanchnicus* and *Bacteroides fragilis* NCTC 9343, respectively). Therefore, gga-miR-222a may be a host regulator that affects H_2_S emission in laying hens by regulating the production of methionine, a sulfur-containing amino acid. In vitro fermentation and bacterial cultivation analysis showed that gga-miR-222a can upregulate the *Odosp_3416* and *BF9343_2953* expression and increase the abundance of *Bacteroides fragilis* NCTC 9343 at the logarithmic growth period (10 h), which resulted in a higher concentration of methionine but lower H_2_S production and soluble sulfide levels in fermentation broth and bacterial culture medium. In previous studies, small RNAs from plants, bacteria, and fungi within the order *Hypocreales* were shown to be ubiquitous in human plasma [38], and the microbiome was observed to play a role in the appropriate regulation of miRNA expression in brain regions implicated in anxiety-like behaviors [39]. The results of these studies demonstrated that miRNAs or small RNAs can function as “regulation messages” between different organisms, and the results of the present study showed that an miRNA of laying hens regulates the physiology of gut bacteria. The concentrations of gut soluble sulfide are positively correlated with the release of H_2_S [40], and observations of decreased H_2_S production and soluble sulfide levels showed that gga-miR-222a can reduce H_2_S emissions in laying hens.

### The host-derived miRNA gga-miR-222a influences H_2_S emission in laying hens

Interestingly, we observed that gga-miR-222a has a positive role in regulating *Odosp_3416* and *BF9343_2953* expression, contrasting with the results of most studies [41, 42] that have indicated miRNAs always inhibit mRNA transcription or directly degrades mRNAs after binding [41, 42]. However, some studies have shown that miRNAs do not always negatively regulate mRNAs [26, 27, 43]. How miRNAs regulate the expression of genes and affect bacterial growth may be dependent on the function of the genes targeted by the miRNA and the site at which miRNAs binds mRNAs. Binding between miRNAs and bacterial transcripts of the 16S rRNA, *yegH*, *RNaseP,* and β-galactosidase genes upregulates their expression and promotes the bacterial growth [31, 32]. In the present study, we observed that gga-miR-222a plays a similar role in the regulation of methionine synthetase gene expression and promoted an increased abundance of bacteria in culture medium, especially in the logarithmic phase. After in situ hybridization, we observed that exogenous gga-miR-222a could be selectively taken up by *Bacteroides fragilis* NCTC 9343, indicating an intracellular cross-regulatory role of gga-miR-222a. However, the increase in bacterial abundance in fermentation broth was not significant except for a slight rise after gga-miR-222a treatment. The reason for this discrepancy may be that the intestinal environment is more complex and there are many interfering factors, such as interactions among various microorganisms, which is why conducting bacterial growth experiment in a pure culture environment was necessary.

## Conclusions

In summary, the results of the present study led to the first identification of host-derived miRNAs in the cecum of laying hens, and the expression profiles of miRNAs were shown to be different between different breeds. It was also demonstrated for the first time that the methionine synthetase genes (*metH*) *Odosp_3416* (expressed by the bacterium *Odoribacter splanchnicus*) and *BF9343_2953* (expressed by the bacterium *Bacteroides fragilis* NCTC 9343) could be targeted by gga-miR-222a and affected the production of H_2_S in laying hens. Additionally, gga-miR-222a could enter *Bacteroides fragilis* NCTC 9343, which increased its abundance in the logarithmic period. Therefore, different profiles of host-derived miRNAs in different breeds of laying hens could affect the production of H_2_S through gene expression regulation in H_2_S production-related bacteria. The regulation of H_2_S production in the cecum of laying hens by host miRNAs such as gga-miR-222a provides the possibility that if these miRNAs could be incorporated into the breeding of laying hens, they could promote the selection of low odor yield and environmentally friendly laying hen breeds.

## Methods

### Animals and feeding

Approximately 100 Hy-line Gray laying hens and 100 Lohmann Pink laying hens were hatched and fed together at a local hatchery to reduce the differences that may be caused by diet, age, weight, and feeding environment. At 28 weeks of age, 30 Hy-line Gray and 30 Lohmann Pink laying hens with similar weights (1.70 ± 0.02 and 1.71 ± 0.02 kg, respectively) were selected from moved into twelve respiration chambers in an environmentally controlled room for daily H_2_S production measurements [29], where 6 replicates (chambers) were set, and five birds were fed in one replicate (chamber). The birds were provided water and the commercial egg-type laying hen diet ad libitum (Table S3), and a 12-h light cycle at 24 °C room temperature management schedule was used. At the end of the experiment, all birds were euthanized by cervical dislocation, and then the cecum was ligated at both sides and removed from the gastrointestinal tract. The contents were aseptically collected into an Eppendorf tube containing Bacterial Protect RNA reagent (Qiagen, Hilden, Germany) at an approximate 1:1 ratio (w/v), immediately frozen in liquid nitrogen and stored at − 80 °C until analysis.

### Animal ethics statement

All animal experiments were approved by the Animal Experimental Committee of South China Agricultural University (SYXK2014-0136). All experimental steps were performed to decrease animal suffering as much as possible. After the experiment, the bodies of laying hens were incinerated.

### The determination of exosomes in the cecum contents

The exosome purification method used in the present study was previously described by Liu et al. Briefly, cecal contents from laying hens were suspended in PBS to 30 mg/mL, centrifuged at 10,000×*g* for 5 min to remove debris and then filtered through a 0.2-μm filter, after which the filtrates were observed using a Thermo Fisher Talos L120C transmission electron microscope (Thermo Fisher Scientific, MA, USA) [26].

### Extraction and analysis of miRNAs from the cecum of laying hens

Total miRNA was extracted using a mirVana™ miRNA Isolation kit (Austin, TX, USA) according to Liu et al. [26]. Briefly, approximately 100 mg of cecal contents were adequately mixed in 600 μL of 1× DPBS, incubated at room temperature for 30 min and then completely homogenized into suspension. Then, 600 μL of acid-phenol:chloroform was added, and the samples were vortexed for 60 s before being then centrifuged for 15 min at 10,000×*g* to separate the organic and aqueous phases. Subsequently, the aqueous phase was recovered, and 1.25 volumes of 100% ethanol were added for miRNA isolation. For each sample, a filter cartridge was placed into one of the collection tubes (supplied by the kit), the sample was pipetted onto a filter and centrifuged for 90 s at 10,000×*g*, and then the flow-through was discarded. Then, the filter was washed with 700 μL of miRNA Wash Solution 1 followed by three washes with 700/500/250 μL of Wash Solution 2/3 (supplied by the kit). Finally, the filter was transferred into a fresh collection tube, and 50 μL of nuclease-free water was applied to the center of the filter. Subsequently, the filter was incubated at room temperature for 10 min before being centrifuged for 5 min at 8000×*g* to recover miRNAs, with the eluate then stored at – 80 °C. Pooled miRNA was prepared by combining equal amounts of extracted miRNA from five birds of the same breed such that each breed was represented by six pooled miRNA samples. miRNA libraries were constructed according to the TruSeq Small RNA Sample Preparation protocol and sequenced with an Illumina HiSeq™ 2500 instrument (Illumina, San Diego, CA, USA). Subsequently, FastQC was used to obtain clean reads from the raw data by removing the joint sequences, low-quality fragments, and sequences < 18 nucleotides (nt) in length. Then, miRDeep2 was used to align the clean sequences to the miRBase database sequences (http://www.mirbase.org/).

### Extraction and analysis of RNA of cecal microbiota

RNA was extracted from cecal content aliquots (200 mg) using an RNeasy® PowerMicrobiome kit (Qiagen, Hilden, Germany) according to the manufacturer’s instructions. Briefly, 200 mg of samples were transferred into PowerBead Tubes. Then, cells were lysed according to the kit manual by adding 650 μL of Solution PM1 with β-mercaptoethanol and 100 μL of phenol/chloroform/isoamyl alcohol and vortexing for 10 minutes at maximum speed using a 24-sample vortex adapter (Kelly Bell, Jiangsu, China). Subsequently, the samples were centrifuged at 13,000×*g* for 1 min at room temperature (15–25 °C), and the supernatants were then transferred to clean 2-mL collection tubes. RNA purification was performed according to the manufacturer’s protocol, and the integrity and quantity of the extracted RNA were measured with a NanoDrop 2000 spectrophotometer (Thermo Scientific, MA, USA) and an Agilent 2100 Bioanalyzer (Agilent Technologies, Palo Alto, CA, USA). Pooled RNA was prepared by homogenization of equal amounts of extracted RNA from five birds of the same breed such that each breed was represented by six pooled RNA samples. The RNA was subjected to standard Illumina library preparation with a TruSeq RNA Sample Prep kit (Illumina, San Diego, CA, USA), and rRNA was depleted using a Ribo-ZeroTM rRNA Removal kit (Epicenter Biotechnologies, Madison, WI, USA). Sequencing was performed on an Illumina HiSeq 2500 sequencer (Illumina, San Diego, CA, USA). Then, the sequences were quality filtered, and poor-quality bases were removed from the raw reads using Cutadapt (v1.9.1). Using a 10-bp sliding window, nucleotides with a mean quality score < 20 were removed, reads with “N” bases (> 10%) and lengths below 75 bp were discarded, and primer sequences and adaptor sequences were also removed. Next, rRNA, tRNA, and host reads were filtered using BWA (v 0.7.5). Putative mRNA reads were then assembled using the Trinity (v2.1.1) de novo assembler. Gene annotation was performed by searching against the protein nonredundant database (NR database), and KEGG (Kyoto Encyclopedia of Genes and Genomes) pathway analysis was conducted for gene function classification. After comparing the transcript profiles between the two breeds, we focused on three pathways related to H_2_S production, including cysteine and methionine metabolism, sulfur metabolism and butyrate metabolism, and the expression of microbial genes associated with these pathways between the two breeds was compared.

### Target prediction of differentially expressed miRNAs

After determining the miRNA profiles in the cecal contents of laying hens, the miRNAs exhibiting significantly different expression between the two breeds were used for target prediction analysis of microbial genes related to H_2_S production exhibiting significantly different expression. The relationships between target bacterial mRNAs and miRNAs were identified using miRanda (http://www.microrna.org). In addition, an analysis of host genome genes targeted by the 10 differentially expressed miRNAs was also conducted with miRanda, and all the target genes were determined to be enriched by KEGG analysis.

### In vitro fermentation experiment

The in vitro fermentation experiment was performed as described by Menke and Steingass [44]. Briefly, thirty 28-week-old Hy-line Gray and thirty Lohmann Pink laying hens were sacrificed, and the ceca were immediately ligated. Then, the cecal contents in the same breed group were pooled and thoroughly mixed with fermentation buffer solution preheated at 39 °C. The fermentation buffer solution was homogenized with 474 mL of deionized water, 237 mL of macroelement solution (per 1000 mL, 5.7 g of Na_2_HPO_4_, 6.2 g of KH_2_PO_4_, and 0.6 g of MgSO_4_·7H_2_O), 237 mL of buffer solution (per 1000 mL, 35.0 g of NaHCO_3_, and 4.0 g of NH_4_HCO_3_), 0.12 mL trace element solution (per 100 mL, 13.2 g of CaCl_2_·2H_2_O, 10.0 g of MnCl_2_·4H_2_O, 1.0 g of CoCl_2_·6H_2_O, and 0.8 g of FeCl_2_·6H_2_O), and 1.22 mL of resazurin and 50 mL of reductant (per 50 mL, 2.0 mL of 1 mol/L NaOH and 335 mg of Na_2_S·9H_2_O). The intestinal content-buffer mixture was blended for 60 s in a blender after the solution was filtered through four layers of surgical gauze, mixed with the fermentation buffer solution at a 1:2 ratio (V/V) and then flushed with CO_2_ at 40 °C to eliminate all the O_2_ in the solution. A corn-soybean basal laying hen diet was used as a substrate for fermentation.

After the air in syringe was eliminated from the head space, approximately 10 mL of fermentation broth (FB) was added to a 100-mL gas syringe with 0.2 g of the substrate. Three different groups were designed for each breed, the blank group (10 mL FB + 0.2 g substrate + 1 mL pure water), the control group (10 mL FB + 0.2 g substrate + 1 mL control mimic at a final concentration of 2 μM) and the treatment group (10 mL FB + 0.2 g substrate + 1 mL gga-miR-222a mimic at a final concentration of 2 μM) (Table S4). The miRNAs used in the present study were purchased from Guangzhou RiboBio Co., Ltd. (Guangzhou, China). Then, these syringes were sealed with clips and placed in incubator set at 42 °C and rotated at 60 rpm for 24 h. At the end of incubation, the syringes were placed on ice to stop fermentation, the gas production was recorded as the volume of head space of the syringe, and the gas was also injected into a gas collection bag for H_2_S analysis. Ten milliliters of fermentation broth was sampled and stored at − 80 °C for chemical analysis. The quantity of H_2_S in the gas sample and the concentrations of soluble sulfide (S^2−^) in the fermentation broth were determined using the methylene blue colorimetric method. Briefly, the adsorption liquid (per 1000 mL, 4.3 g of CdSO_4_·8H_2_O, 0.3 g of NaOH, and 10 g of ammonium polyvinyl phosphate) was mixed with gas (10 mL of adsorption liquid) and fermentation broth (adsorption liquid:fermentation broth, V/V = 9:1), after which procedures were followed as described in previous studies [45, 46]. The pH value was determined using a pH meter (INESA Scientific Instrument, Shanghai, China) [29], the concentration of sulfate radicals (SO_4_^2−^) was determined using the turbidimetric method [29], the concentrations of VFAs were determined using high-performance liquid chromatography [29], and the concentration of methionine in the fermentation broth as tested using an automatic amino acid analyzer (Sykam, Munich, Germany). For the latter analysis, 1 mL of fermentation broth was mixed with 10 mL of hydrochloric acid solution (6 mol/L) followed by 3–4 drops of phenol before being incubated on ice for 3–5 min. Subsequently, the sample was flushed with nitrogen to remove oxygen before incubated in an air oven at 110 °C for 22 h. After cooling at room temperature, the solution was brought to a final volume of 50 mL after filtering using filter paper. The 15 mL of filtered solution was dried at 40–50 °C, and the deposition was washed with deionized water twice and dried again. Then, the precipitate was resuspended in 1 mL of a 0.02-mol/L hydrochloric acid solution, and after filtering through a 0.22-μm membrane, the methionine concentration was determined with an automatic amino acid analyzer.

### Bacterial abundance and gene expression in fermentation broth

Following the manufacturer’s instructions, a QIAamp PowerFecal DNA kit (Qiagen, Hilden, Germany) was used to extract DNA from the precipitate (approximately 200 mg) collected from 1 mL of fermentation broth centrifuged at 20,000×*g* for 1 min at 4 °C. Following the manufacturer’s instructions, an RNeasy® PowerMicrobiome™ kit (Qiagen, Hilden, Germany) was used to extract RNA from the precipitate (approximately 200–250 mg) collected from 1–2 mL of fermentation broth centrifuged at 20,000×*g* for 1 min at 4 °C.

DNA was used to quantify the relative abundances of *Odoribacter splanchnicus* and *Bacteroides fragilis* NCTC 9343. Briefly, primers for these two bacteria were designed using Primer 3, and the genome sequences of the two bacteria were referenced for 16S rRNA sequencing on the NCBI website. The details of the primers are shown in Table S5, and the bacterial 16S rRNA primers were referenced in a previous study [47]. Q-PCR was used to confirm the relative abundances of the two bacteria, and the q-PCR reaction steps followed the protocol for the SYBR® Green PCR kit (SYBR, Japan) (Table S6). The relative abundances of the two bacteria were calculated using the 2^ΔCt^ method, where ΔCt represents the difference in the Ct value for the 16S rRNA gene minus that for the reference genes [48].

Extracted RNA was reverse transcribed into cDNA using a PrimeScript™ RT reagent kit (TaKaRa, Kusatsu, Japan), and the resulting cDNA was used to quantify the expression of *Odosp_3416* and *BF9343_2953.* The primers were designed using the NCBI website using the complete bacterial 16S rRNA gene as the reference gene (Table S5). Q-PCR was used to confirm the relative expression levels of the two genes, and the q-PCR reaction steps followed the protocol for the SYBR® Green PCR kit (SYBR, Japan) with slight modification (Table S7). The relative expression levels of the two genes were calculated using the 2^△Ct^ method, where ΔCt represents the difference in the Ct value for the 16S rRNA gene minus that for the reference genes [48].

### In vitro bacterial growth measurements

The anaerobic bacterium *Bacteroides fragilis* NCTC9343 was cultured at 37 °C by inoculating 40-mL aliquots of anaerobic basal medium (Becton Dickinson and Company, Lincoln Park, IL, USA) and then grown anaerobically in an anaerobic chamber (Mitsubishi Gas Chemical Company, Inc. Tokyo, Japan). gga-miR-222a and the control mimic were added to the culture at a concentration of 2 μM (RiboBio, Guangzhou, China). Growth was monitored as absorbance at 600 nm once per hour for up to 24 h with a spectrophotometer. The cultured bacterial cells were collected after 10 h and used to assess *BF9343_2953* expression using the *Bacteroides fragilis* 16S rRNA gene as the reference gene. The concentrations of methionine in culture medium at 10 h were tested as described above.

### In situ hybridization detection of gga-miR-222a uptake

*Bacteroides fragilis* NCTC9343 cells were centrifuged at 12,000×*g* and washed twice with ice-cold PBS. Then, the cells were fixed in 4% PFA/0.25% glutaraldehyde. A 5′-DIG and 3′-DIG dual-labeled probe for gga-miR-222a was used for in situ hybridization, and detection of gga-miR-222a uptake by bacteria was assessed using a Thermo Fisher Talos L120C transmission electron microscope Thermo (Fisher Scientific, MA, USA).

### Statistical analysis

Comparisons of fermentation incubation indexes; the relative abundances of miRNA, bacteria, and genes; gas production levels; H_2_S production; and growth curves were examined by analysis of variance (ANOVA) with Statistical Package for the Social Sciences (SPSS), version 22.0. Significant differences between the means were determined by Tukey’s test.

### Differences were considered significant at *P* < 0.05

The metatranscriptomic results of each sample were analyzed using HTSeq using the union model, and the number of genes at different expression levels and the expression levels of individual genes were statistically analyzed. In general, an FPKM value of 0.1 or 1 was used as the threshold for determining whether genes are expressed. DESeq was used to normalize the read counts from analysis of gene expression levels [49].

miRNA expression levels were calculated with the TPM formula [normalization read counts = ((readCount × 1,000,000)/libsize)], where libsize is the sum of the read count of all miRNAs.

## Supplementary Information

**Additional file 1: Table S1**. miRNA sequencing information. **Table S2** Metatranscriptomic sequencing information, where (A) shows the metatranscriptomic sequencing information and (B) shows the total transcript length after splicing. **Table S3** Diet composition and nutrient levels. **Table S4** Group details. **Table S5** Primer information for bacterial abundance and gene expression. **Table S6** Quantitative PCR thermal cycle programs for bacteria. **Table S7** Quantitative PCR thermal cycle programs for genes.

**Additional file 2: Fig. S1**. KEGG pathway annotation of chicken genome target genes of 10 significantly expressed miRNAs.

**Additional file 3: Fig. S2**. Metatranscriptomic annotation of the top 30 KEGG metabolic pathways.

**Additional file 4: Fig. S3**. Relative abundances of *Odoribacter splanchnicus* and *Bacteroides fragilis* NCTC9343 in the intestines of Lohmann and Hy-line hens. * Indicates a significant difference between different bacteria, and significant differences between the means were determined by Tukey’s test (*P* < 0.05).

## Data Availability

The miRNA and metatranscriptomic sequence data are deposited in the NCBI Sequence Read Archive (SRA) database (PRJNA665380 and PRJNA666118)

## Abbreviations

H_2_S: Hydrogen sulfide
SO_4_^2−^: Sulfate radical
S^2−^: Soluble sulfide
SRB: Sulfate-reducing bacteria
VFAs: Volatile fatty acids
