# Supplementary material for: Breed differences in the expression levels of gga-miR-222a in laying hens influenced H2S production by regulating methionine synthase genes in gut bacteria
Source: Microbiome. 2021 Aug 25;9:177. doi: 10.1186/s40168-021-01098-7 (PMC8390279; doi:10.1186/s40168-021-01098-7)
Supplement: Supplementary file 2 — Additional file 1: Table S1. miRNA sequencing information. Table S2 Metatranscriptomic sequencing information, where (A) shows the metatranscriptomic sequencing information and (B) shows the total transcript length after splicing. Table S3 Diet composition and nutrient levels. Table S4 Group details. Table S5 Primer information for bacterial abundance and gene expression. Table S6 Quantitative PCR thermal cycle programs for bacteria. Table S7 Quantitative PCR thermal cycle programs for genes. [file 40168_2021_1098_MOESM2_ESM.docx]

Table S1. The sequencing information details of miRNA

| **Sample** | **Raw Reads** | **Clean Reads** | **GC Content %** | **Q20 bases %** | **Q30 bases %** | **Total mapped（% of clean reads）** |
| --- | --- | --- | --- | --- | --- | --- |
| L1 | 13,884,925 | 13,208,044 | 53.24 | 98.61 | 97.29 | 1577866（11.95%） |
| L2 | 13,736,575 | 13,211,959 | 53.33 | 98.62 | 97.31 | 918643（6.95%） |
| L3 | 13,833,075 | 13,209,714 | 53.27 | 98.62 | 97.3 | 1600213（12.11%） |
| L4 | 18,861,125 | 14,395,728 | 52.69 | 98.41 | 96.93 | 5709449（39.66%） |
| L5 | 14,233,750 | 13,207,911 | 54.97 | 98.47 | 97 | 3101933（23.49%） |
| L6 | 13,882,800 | 13,209,585 | 53.36 | 98.55 | 97.18 | 1691444（12.8%） |
| H1 | 13,873,875 | 13,204,692 | 53.43 | 98.58 | 97.22 | 1765322（13.37%） |
| H2 | 13,707,675 | 13,207,335 | 52.7 | 98.59 | 97.26 | 671728（5.09%） |
| H3 | 13,685,250 | 13,209,570 | 53.98 | 98.61 | 97.3 | 1186191（8.98%） |
| H4 | 13,755,050 | 13,204,285 | 53.02 | 98.56 | 97.2 | 1559409（11.81%） |
| H5 | 14,058,925 | 13,210,526 | 52.93 | 98.6 | 97.27 | 1728119（13.08%） |
| H6 | 13,802,225 | 13,207,757 | 53.66 | 98.61 | 97.28 | 1398750（10.59%） |

L means Lohmann and H means Hy-line. Q20 means the percentage of bases with a Phred value greater than 20, and Q30 means the percentage of bases with a Phred value greater than 30. Total mapped means the number of sequences mapped to the genes of the host.

Table S2A. The sequencing information details of metatranscriptomic

| **Sample** | **Raw Reads** | **Clean Reads** | **Clean Bases** | **Q20 %** | **Q30 %** | **GC Content %** | **Total mapped（% of clean reads）** |
| --- | --- | --- | --- | --- | --- | --- | --- |
| L1 | 34758534 | 34100304 | 5.12G | 97.59 | 93.13 | 49.28 | 26310424(77.16%) |
| L2 | 38750340 | 37957512 | 5.69G | 97.65 | 93.32 | 48.87 | 27329534(72.00%) |
| L3 | 39773026 | 38928974 | 5.84G | 97.68 | 93.39 | 49.3 | 28973762(74.43%) |
| L4 | 47250672 | 46162396 | 6.92G | 97.65 | 93.38 | 50.05 | 34420886(74.56%) |
| L5 | 39830766 | 39155558 | 5.87G | 97.42 | 92.79 | 48.07 | 27330614(69.80%) |
| L6 | 41090622 | 39647620 | 5.95G | 97.85 | 93.78 | 48.85 | 30641922(77.29%) |
| H1 | 39631504 | 38654950 | 5.8G | 97.4 | 92.93 | 52.56 | 26418178(68.34%) |
| H2 | 47219800 | 45976710 | 6.9G | 97.9 | 93.92 | 50.92 | 36774628(79.99%) |
| H3 | 39162438 | 38122898 | 5.72G | 97.7 | 93.54 | 53.05 | 25109578(65.86%) |
| H4 | 39341228 | 38449368 | 5.77G | 97.68 | 93.41 | 49.85 | 26721834(69.50%) |
| H5 | 48453278 | 47179902 | 7.08G | 97.45 | 93.1 | 50.02 | 29175334(71.34%) |
| H6 | 47518656 | 46468862 | 6.97G | 97.29 | 92.8 | 49.68 | 27378952(71.36%) |

L means Lohmann and H means Hy-line. Q20 means the percentage of bases with a Phred value greater than 20, and Q30 means the percentage of bases with a Phred value greater than 30. Total mapped means the number of sequences mapped to the genes of the flora.

Table S2B. The total transcript length after splicing

| **Transcript length interval** | 300-500bp | 500-1kbp | 1k-2kbp | >2kbp | Total |
| --- | --- | --- | --- | --- | --- |
| **Number of transcripts** | 821212 | 510314 | 209133 | 89319 | 1629978 |

Table S3. Diet composition and nutrient levels

| **Ingredients** | **Content (%)** | **Nutrient levels** | **Content** |
| --- | --- | --- | --- |
| Maize meal | 62 | ME (MJ/kg) | 11.13 |
| Soybean meal | 26 | CP (%) | 16.2 |
| Soybean oil | 1 | Ca (%) | 3.8 |
| Limestone | 8 | TP (%) | 0.45 |
| Monocalcium phosphate | 1.54 | AP (%) | 0.32 |
| Salt | 0.3 | Lys (%) | 0.8 |
| DL-Met | 0.16 | Met (%) | 0.38 |
| Premix ^1^ | 1 |  |  |
| Total | 100 |  |  |

^1^ The premix provided the following per kg of diets: Mn 60mg, Cu 8mg, Zn 80mg, Fe 60mg, I 0.35mg, Se 0.3mg, VA9000IU, VD3 1600 IU, VE 5 IU, VK 0.5mg, VB12 0.004mg, Biotin 0.1mg, Folate 0.25mg, Niacin 20mg, Pantothenate 25mg, VB6 3mg, VB2 25mg, Choline 500mg.

Table S4. The group details

| **Groups** | **FB (mL)** | **substrate (g)** | **sterile water (mL)** | **miRNA control (mL)** | **miRNA (mL)** |
| --- | --- | --- | --- | --- | --- |
|  |  |  |  |  |  |
| LB | 9 | 0.2 | 1 |  |  |
| LC | 9 | 0.2 |  | 1 |  |
| LT | 9 | 0.2 |  |  | 1 |
| HB | 9 | 0.2 | 1 |  |  |
| HC | 9 | 0.2 |  | 1 |  |
| HT | 9 | 0.2 |  |  | 1 |

Note: The miRNA product is freeze-dried powder, diluted into 20 μm stock solution before using. The *in vitro* fermentation reaction volume is 10mL. FB means fermentation broth.

Table S5. Primer information for bacteria abundance and gene expression

| **Gene** | **Primer sequences** |
| --- | --- |
| *Odoribacter splanchnicus* 16S rRNA | F：TGAGGTGTCGGGTTAAGTCC |
|  | R：TGTGTAGCCCTGGGTGTAAG |
| *Bacteroides fragilis* NCTC 9343 16S rRNA | F：GCCAAGTAGCGTGAAGGATG |
|  | R：TGAGCCGCAAACTTTCACAA |
| *Odosp_3416* | F：TGTCCCAAGGCTTACGGAAC |
|  | R：CGGGCACATAAGCTTTCACG |
| *BF9343_2953* | F：TCGGCTGATGAGTCCGAAAT |
|  | R：TCACGCTTCATCATGCCACT |
| Bacterial 16S rRNA | F：CGGCAACGAGCGCAACCC |
|  | R：CCATTGTAGCACGTGTGTAGCC |

Table S6. Quantitative PCR thermal cycle programs of bacteria

| **Programs** | **Thermal cycle programs** |
| --- | --- |
| Denaturing | 95℃ 2min |
| Denaturation | 95℃ 5s |
| Annealing | 60℃ 30s |
| Extension | 72°C 25 s |
| Cycles | 39 |
| Final extension | 72°C 10 min |
| dissolution curve | 60-95°C |

Table S7. Quantitative PCR thermal cycle programs of genes

| **Programs** | **Thermal cycle programs** |
| --- | --- |
| Denaturing | 95℃ 3min |
| Denaturation | 95℃ 10s |
| Annealing | 60℃ 30s |
| Extension | 72°C 40 s |
| Cycles | 40 |
| Final extension | 72°C 10 min |
| dissolution curve | 60-95°C |
