# Supplementary figures and images for: Breed differences in the expression levels of gga-miR-222a in laying hens influenced H2S production by regulating methionine synthase genes in gut bacteria
Source: Microbiome. 2021 Aug 25;9:177. doi: 10.1186/s40168-021-01098-7 (PMC8390279; doi:10.1186/s40168-021-01098-7)

Statistics of Pathway Enrichment

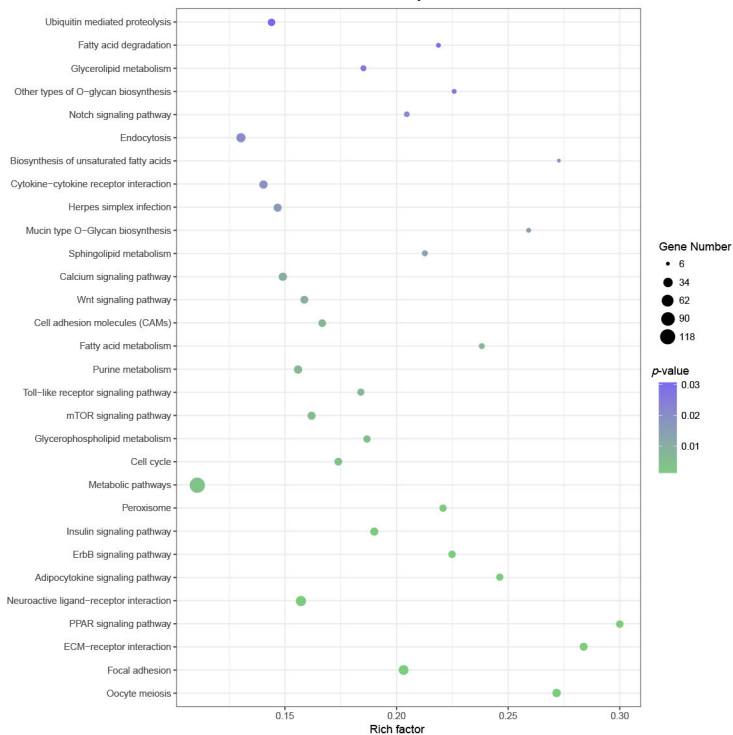

Supplement: Supplementary file 3 — Additional file 2: Fig. S1. KEGG pathway annotation of chicken genome target genes of 10 significantly expressed miRNAs. [file 40168_2021_1098_MOESM3_ESM.pdf]

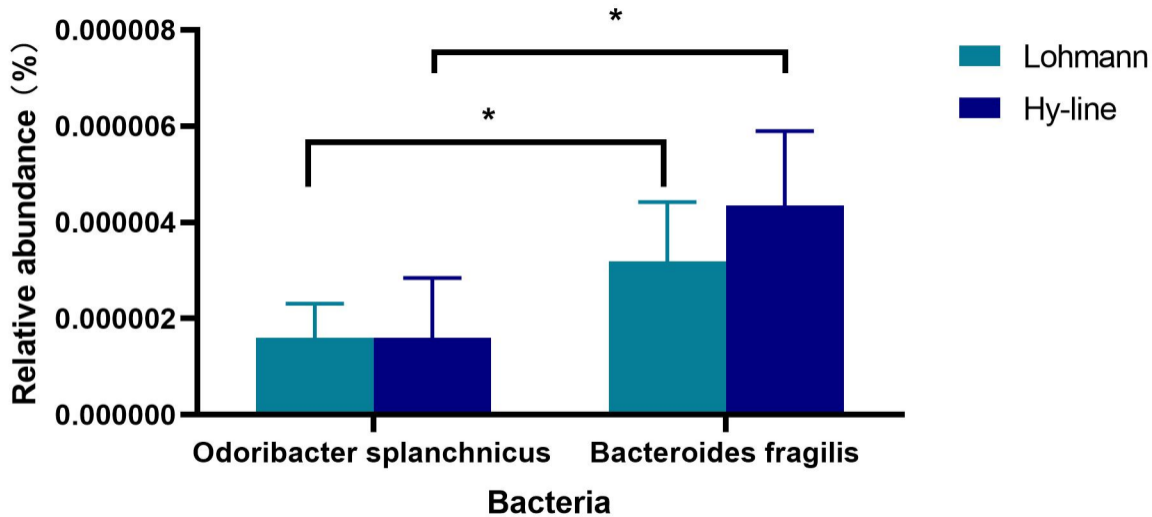

Supplement: Supplementary file 5 — Additional file 4: Fig. S3. Relative abundances of Odoribacter splanchnicus and Bacteroides fragilis NCTC9343 in the intestines of Lohmann and Hy-line hens. * Indicates a significant difference between different bacteria, and significant differences between the means were determined by Tukey’s test (P < 0.05). [file 40168_2021_1098_MOESM5_ESM.pdf]
