# Supplementary material for: Breed differences in the expression levels of gga-miR-222a in laying hens influenced H2S production by regulating methionine synthase genes in gut bacteria
Source: Microbiome. 2021 Aug 25;9:177. doi: 10.1186/s40168-021-01098-7 (PMC8390279; doi:10.1186/s40168-021-01098-7)
Supplement: Supplementary file 4 — Additional file 3: Fig. S2. Metatranscriptomic annotation of the top 30 KEGG metabolic pathways. [file 40168_2021_1098_MOESM4_ESM.pdf]

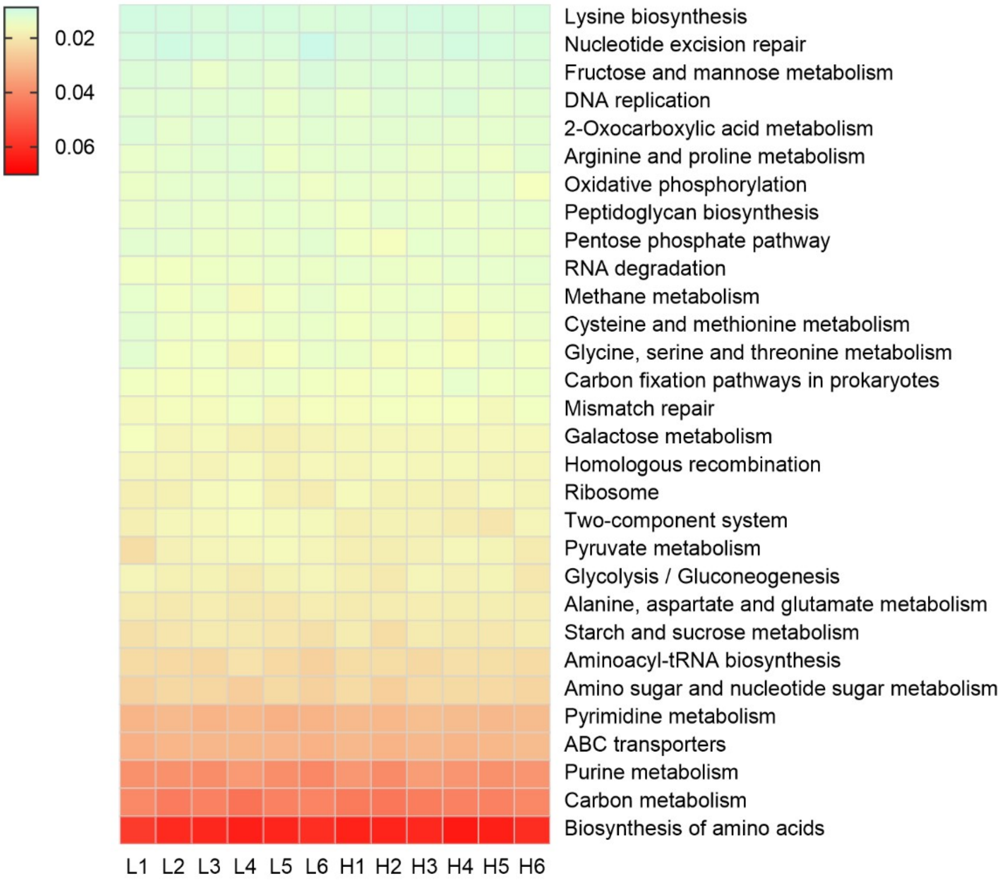
0.02
0.04
0.06
Lysine biosynthesis
Nucleotide excision repair
Fructose and mannose metabolism
DNA replication
2-Oxocarboxylic acid metabolism
Arginine and proline metabolism
Oxidative phosphorylation
Peptidoglycan biosynthesis
Pentose phosphate pathway
RNA degradation
Methane metabolism
Cysteine and methionine metabolism
Glycine, serine and threonine metabolism
Carbon fixation pathways in prokaryotes
Mismatch repair
Galactose metabolism
Homologous recombination
Ribosome
Two-component system
Pyruvate metabolism
Glycolysis / Gluconeogenesis
Alanine, aspartate and glutamate metabolism
Starch and sucrose metabolism
Aminoacyl-tRNA biosynthesis
Amino sugar and nucleotide sugar metabolism
Pyrimidine metabolism
ABC transporters
Purine metabolism
Carbon metabolism
Biosynthesis of amino acids
L1
L2
L3
L4
L5
L6
H1
H2
H3
H4
H5
H6
